# Hydrogel Microparticles as Sensors for Specific Adhesion: Case Studies on Antibody Detection and Soil Release Polymers

**DOI:** 10.3390/gels3030031

**Published:** 2017-08-08

**Authors:** Alexander Klaus Strzelczyk, Hanqing Wang, Andreas Lindhorst, Johannes Waschke, Tilo Pompe, Christian Kropf, Benoit Luneau, Stephan Schmidt

**Affiliations:** 1 Institute for Organic and Macromolecular Chemistry, Heinrich-Heine-Universität, Universitätsstrasse 1, 40225 Düsseldorf, Germany; Alexander.Strzelczyk@uni-duesseldorf.de (A.K.S.); hanqing.wang@hhu.de (H.W.); 2 Institute for Biochemistry, Universität Leipzig, Johannisallee 21-23, 04103 Leipzig, Germany; Andreas_Lindhorst@web.de (A.L.); jowaschke@cbs.mpg.de (J.W.); tilo.pompe@uni-leipzig.de (T.P.); 3 Henkel AG & Co KGaA, Henkelstr 67, D-40589 Düsseldorf, Germany; christian.kropf@henkel.com (C.K.); benoit.luneau@henkel.com (B.L.)

**Keywords:** biomimetic hydrogel, biointerface, elastic solids, contact mechanics, poly(ethylene glycol) (PEG), soft colloidal probe, reflection interference contrast microscopy (RICM)

## Abstract

Adhesive processes in aqueous media play a crucial role in nature and are important for many technological processes. However, direct quantification of adhesion still requires expensive instrumentation while their sample throughput is rather small. Here we present a fast, and easily applicable method on quantifying adhesion energy in water based on interferometric measurement of polymer microgel contact areas with functionalized glass slides and evaluation via the Johnson–Kendall–Roberts (JKR) model. The advantage of the method is that the microgel matrix can be easily adapted to reconstruct various biological or technological adhesion processes. Here we study the suitability of the new adhesion method with two relevant examples: (1) antibody detection and (2) soil release polymers. The measurement of adhesion energy provides direct insights on the presence of antibodies showing that the method can be generally used for biomolecule detection. As a relevant example of adhesion in technology, the antiadhesive properties of soil release polymers used in today’s laundry products are investigated. Here the measurement of adhesion energy provides direct insights into the relation between polymer composition and soil release activity. Overall, the work shows that polymer hydrogel particles can be used as versatile adhesion sensors to investigate a broad range of adhesion processes in aqueous media.

## 1. Introduction

**Figure 1:**
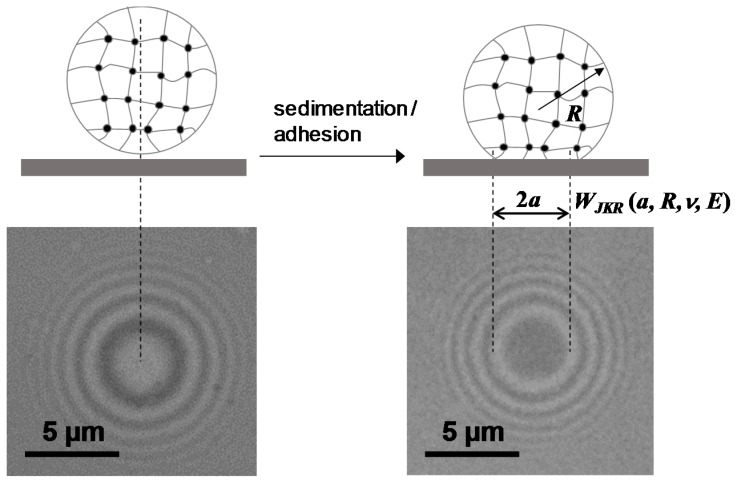
Principle of the Johnson-Kendall-Roberts (JKR) adhesion measurements with colloidal probes and typical reflection interference contrast microscopy (RICM) images (**bottom**) right before and after SCP adhesion. The dark area in the middle signifies the soft colloidal probe (SCP) contact area with the solid support.

Adhesive processes in aqueous environment are of great importance, e.g., in biology where crucial cellular functions are controlled by biological interfaces decorated with carbohydrates, proteins or lipids. Controlling adhesive interaction under water is also very important in technology, e.g., mussel adhesion on surfaces of ships as well as for cleaning processes or controlling the haptic sensation of surfaces [1,2]. On the molecular level, adhesion phenomena are typically indirectly studied using methods like quartz crystal microbalance, surface plasmon resonance or fluorescence microscopy [3,4]. These methods simply relate adhesion to the adsorbed amount of the molecules of interest. However, without direct measurement of adhesive interactions these methods provide only limited insight into the underlying mechanisms. Alternatively, direct force measurements by atomic force microscopy (AFM), surface force apparatus are suitable to directly study adhesion phenomena between material surfaces [5,6,7]. In addition, such adhesion assays offer information on the nature of the interaction; for example, discrimination between specific and non-specific binding. However, these methods are often expensive, not easy to operate and slow when compared to other analytic techniques. Therefore, we have developed a fast, easy-to-use method that can be adapted to investigate and quantify a broad range of adhesion phenomena in aqueous environment. The method uses soft hydrogel microparticles (soft colloidal probes, SCPs) as adhesion sensors on glass surfaces functionalized with a suitable binding partner. The working principle of adhesion measurements based on hydrogel SCPs is schematically explained in Figure 1. When adhering to a functionalized glass coverslip surface, the SCPs undergo mechanical deformation due to their soft, gel-like structure [8,9]. The mechanical deformation can be related to the adhesion energy of the SCP with the surface by means of the Johnson–Kendall–Roberts (JKR) model of adhesion [10]:

(1) $$W_{adh}=\frac{E_{eff}\;a^{3}}{6\pi \;R^{2}}$$

where *a* is the radius of contact, *R* radius of the SCP and *E_eff_* = [4*E*/3(1 − *ν*^2^)] its effective elastic modulus, with *ν* the Poisson ratio and *E* the elastic modulus of the SCP. Technically, the read-out of adhesion energies is straightforward and fast. First, *E* can be determined batch-wise for a large amount of SCPs by force-indentation measurements using an atomic force microscope. Assuming volume conservation *ν* can be taken as 0.5 in order to determine *W_adh_*. SCPs of well-known mechanical properties are then subject to adhesion on various sample surfaces where *a* and *R* can be directly measured by an interferometric technique (reflection interference contrast microscopy, RICM) [11]. An additional advantage of this method is that PEG hydrogel particles as adhesion sensors show reduced non-specific binding to material surfaces and impurities due to the strong hydration of the PEG matrix. In addition, by functionalizing the PEG matrix with various molecules, including proteins [12], peptides [13] or carbohydrates [14], the assay can be adapted in a straightforward fashion to study adhesion phenomena in different contexts and application areas. Due to their biomimetic properties, SCPs have recently been successfully used as AFM probes to investigate interactions of cells [15] and biomaterial surfaces [16,17].

Here we now focus on testing the applicability of the method in two applied areas, (1) detection of antibodies and (2) investigation of soil release processes of polymeric detergent additives. Given the commercial importance of these two areas, new ways of studying the underlying molecular interaction measurements is important. For instance, antibody detection is of great importance in medical diagnostics. Increased levels of antibodies in blood generally indicate exposure to certain antigens. Medical diagnostics routinely quantifies antibody concentrations in blood, which can be related to the presence of certain pathogenic antigens such as human immunodeficiency virus (HIV), measles or hepatitis [18]. More recent developments in antibody detection are directed towards diagnosis of cancer or autoimmune diseases [19,20]. The enzyme linked immunosorbent assay (ELISA) is the most commonly used antibody detection method due to its comparatively high sensitivity and selectivity. However, extensive cleaning procedures are required in order to achieve sufficiently high selectivity, which often hampers routine application of ELISA in medical diagnostics due to economic reasons. Therefore, here we aim at antibody detection by measuring the adhesion between antigen-coated hydrogel SCPs that capture antibodies and surfaces functionalized with protein A, an antibody binding protein. The potential advantage of this approach is that the extensive cleaning procedures usually required in ELISA would be significantly reduced. Sensitivity and selectivity are shown by determining the detection limit of the method as well as performing the assay in presence of interfering protein impurities. In the second application of the SCP adhesion assay, we investigate the ‘soil release’ activity of various polymer samples. Soil release polymers are often present in modern consumer detergent for fabric cleaning. They are supposed to fulfill two basic functions: (1) hindering redeposition of the soil (e.g., fats) from the washing solution to the fabric during washing, (2) formation of a protective coating on the fabric right after washing to enhance release of acquired hydrophobic soil in the next washing step. For both processes, it could be stated that soil release polymers act as antiadhesives. There is a range of polymers that have been empirically identified as soil release active. However, the soil release process and the underlying working principle of the active polymers so far have not been studied systematically. Therefore, we set out to mimic the adhesion of cotton fabrics on oily materials using functionalized SCPs and then quantitatively study the antiadhesive properties of various soil release polymers.

## 2. Results and Discussion

### 2.1. Synthesis and Functionalization of SCPs with Adhesion Molecules

**Figure 2:**
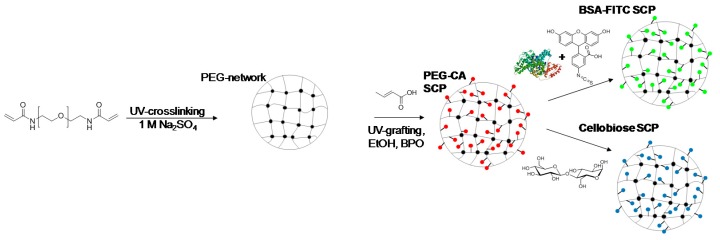
Sythetic route toward bovine serum albumin-fluoresceine isothiocyanate (BSA-FTIC) SCPs and cellubiose SCPs based on PEG-dAAm microgels.

As outlined in Figure 2, SCPs with a hydrogel matrix were prepared by crosslinking of poly(ethylene glycol) (8 kDa) diacrylamide (PEG_8kDa_-dAAm) macromonomer droplets in 1 M Na_2_SO_4_ in water following previously established protocols [8]. For the two adhesion assays, antibody detection and soil release polymer characterization, two types of SCPs were prepared: antigen and cellobiose functionalized SCPs. In order to introduce coupling groups for the antigen and cellobiose for the adhesion assay, crotonic acid (CA) was grafted in the PEG-dAAm network under UV irradiation using benzophenone as active photophore [21,22]. Accordingly, PEG-CA SCPs with a CA functionalization degree on the order of 120 µmol CA per 1 g PEG-CA were obtained as measured via Toluidine blue O (TBO) titration.

**Figure 3:**
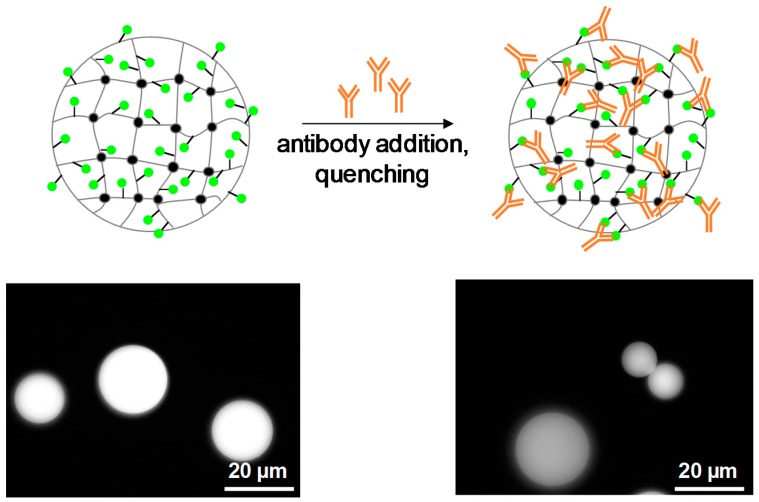
Fluorescence microscopy of BSA-FITC SCPs without addition of antibodies (**left**) and after addition of antibodies (**right**). Reduction on fluorescence intensity is due to quenching upon antibody binding and signifies specific interaction of the antibody with the SCPs.

**Figure 4:**
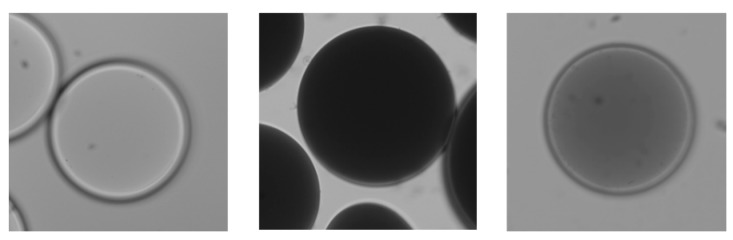
Toluidine blue (TBO) stained PEG SCPs before functionalization with crotonic acid (CA) (**left**). After CA functionalization PEG-CA SCPs bind TBO and acquire a dark color (**middle**). Reduced take-up of TBO after functionalization of CA groups with cellobiose (**right**).

For the preparation of antigen functionalized systems, fluoresceinisothiocyanate (FITC) was chosen as a model antigen. In order to functionalize SCPs with FITC, we used bovine serum albumin (BSA) as a carrier. First, BSA was coupled to the SCPs by activating the CA groups with ethyl dimethylaminopropyl carbodiimide (EDC) and *N*-hydroxysuccinimide (NHS) before binding the protein to the SCPs. Then the SCPs were cleaned and directly reacted with FITC that binds to the nucleophilic amino acid side chains of BSA, resulting in BSA-FITC SCPs. Successful functionalization of the SCPs could be readily confirmed by fluorescence microscopy detecting the presence of FITC on the SCPs (Figure 3). For the preparation of cellobiose-functionalized systems mimicking cotton fabrics, PEG-CA SCPs were reacted with the carbodiimide, activating the carboxylic acid groups to then form an ester with cellobiose. The degree of cellobiose functionalization was measured by an additional titration step with toluidine blue that essentially yielded the reduction of carboxylic acids due to esterification upon cellobiose coupling. The conversion was on the order of 90%, i.e., functionalization degrees on the order of 100 µmol g^−1^ were achieved. Taking into account the molecular weight of PEG-dAAm chains of 8000 kDa, this means that circa 0.8 cellobiose units per macromonomer were bound to the SCPs. Successful functionalization could be additionally confirmed by optical microscopy, where the TBO-labeled PEG-CA SCPs show a strong blue color that becomes significantly less intense in the case of cellobiose SCPs (Figure 4), indicating cellobiose functionalization. The elastic modulus of the final SCPs was determined by AFM force-indentation measurements (Figure S1) and evaluation with a recently introduced model [23]. This model considers that the SCPs deform at the indenter site and the contact site with the solid substrate. The model further assumes linear elasticity, which is justified by the fact that the deformation during force indentation measurements does not exceed 0.2% with respect to the particle’s diameter. Both the BSA-FITC SCPs and cellobiose SCPs showed an elastic modulus of around 60 kPa.

### 2.2. Detection of Antibodies with a Combined SCP Pull-Down and Adhesion Assay

**Figure 5:**
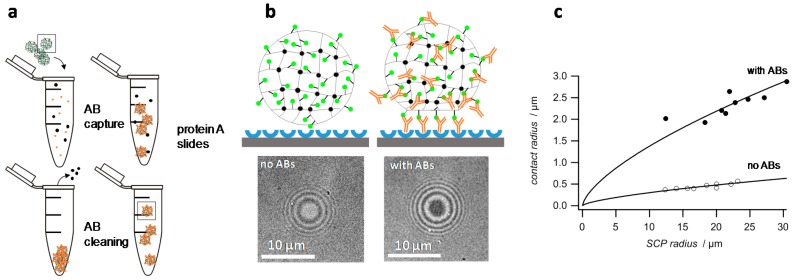
Procedure of the SCP adhesion assay. First BSA-FITC SCPs are incubated in antibody solution. Then they are cleaned by centrifugation and washing (**a**). Next, the SCPs adhesion is measured on protein A slided (**b**). The micrographs show images of an untreated (**left**) and antibody treated BSA-FITC SCP (**right**). After measurement of the contact area, the JKR plots reveal the adhesion energies of the SCPs (**c**). Note that drawings in (**b**) are not to scale and are presenting an idealized orientation of the binding partners for clarity.

The FITC represent the model antigen in the adhesion assay with BSA-FITC SCPs. The overall concept is to capture the antibody from solution with the SCPs (pull-down assay) and then to detect the presence of the antibody by the actual SCP adhesion measurement (Figure 5). The FITC antibody used in this work belongs to one of the most important antibody subclasses-IgG (immunglobulin G)-as most clinic applications with antibodies rely on this subtype. IgG antibodies are composed of two identical heavy chains and two identical light chains which form the characteristic Y-shaped quaternary structure by intramolecular disulfide bonding. The two antigen binding sites are each formed by a light and heavy chain. The third specific binding site is formed only by the heavy chain, the so-called Fc-region at the base of the “Y”. The specific affinity to the respective antigens is due to the large variability of amino acid sequences of the light chain, whereas the heavy chains are always identical for each antibody subtype [18]. Therefore, all antibodies belonging to the IgG subtype can be detected by specific binding at the Fc site. Here we use protein A as specifically interacting species binding to the Fc region in order to detect the antibody by means of adhesion. Additionally, for a preliminary test, the specific binding of the antibody to BSA-FITC SCPs can be visualized by fluorescence microscopy. This is due to fluorescence quenching of FITC when binding to an antibody [24]. The fluorescence microcopy measurements confirm binding of the antibody to the FITC-BSA SCPs, as can be seen by the ~30% reduction in fluorescence intensity (Figure 4). The reduction in intensity at the SCPs is lower as compared to experiments with FITC in solution [24], possibly because of a dense functionalization of BSA with FTIC.

Having confirmed the specific interactions of the IgG antibody with the BSA-FTIC SCPs, the adhesion energies of SCPs were measured on a protein A slide as a means to detect the antibodies (Figure 5b). In a first study, the BSA-FITC SCPs were incubated for two hours in a 0.1 mg mL^−1^ solution of the antibody in phosphate buffered saline (PBS). Next, the SCPs were centrifuged and washed with PBS three times to remove unbound antibodies. The protein A surface was prepared simply by allowing physisorption of protein A on the cleaned hydrophilic glass coverslips followed by physisorption of BSA on the coverslip to reduce non-specific binding sites. Next, the actual adhesion measurement was conducted by adding the SCPs to the coverslips in PBS. As expected, the contact areas of the SCPs with the protein A surface were significantly larger for SCPs treated with the antibody as compared to the negative control without antibodies due to the binding of the Fc-region with the protein A slides (Figure 5b). The contact areas versus SCP radius data could be fitted by equation 1 in order to obtain the adhesion energies of the SCPs (Figure 5c).

**Figure 6:**
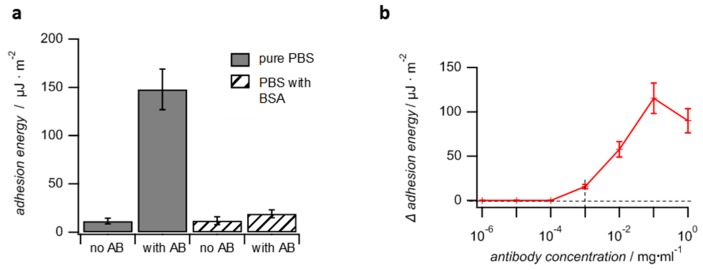
Results for SCPs adhesion assays for antibody detection. (**a**) Adhesion energies of BSA-FITC SCPs after incubation with antibodies (AB) on protein A slides. Measurements without antibody treatment were conducted as negative control. Measurements in presence and absence of 50 mg mL^−1^ BSA were conducted to investigate the selectivity of the method. (**b**) Measurement of BSA-FITC SCPs treated in different concentrations of antibody solution show that the detection limit is on the order of 1 µg mL^−1^ antibody.

The adhesion energy of antibody-treated SCPs was roughly 20 times larger as compared to SCPs without antibody (Figure 6a) Next, the antibody detection limit was studied by charging the SCPs in solutions with antibody at concentrations ranging from 1 ng to 1 mg (Figure 6b). For antibody concentrations below 1 µg mL^−1^, the adhesion energies did not increase compared to the negative control. Therefore, for the SCPs system established here, the detection limit is approximately 1 µg mL^−1^. For comparison, the majority of commercial ELISA kits for protein analysis claim detection limits in the sub ng mL^−1^ range. Advanced ELISA methods may even reach detection limits in the lower pg mL^−1^ regime [25,26,27]. In unfavorable cases of low affinity between antibody and antigen in the micromolar range, the ELISA detection limit is increased up to 100 ng mL^−1^ [28]. Nevertheless, with a detection limit of 1 µg mL^−1^, the SCP assay proved to be inferior to typical ELISA assays. The main reason could be the requirement of having both the protein A and the antibody at the glass surface and the SCP orientated in the right direction, so that they face each other in order to bind the antibodies Fc site. In case of the protein A surface, optimizing the orientation should be less important. The protein possesses five homologous binding sites for the FC-region of which a sufficient number is always likely to be accessible even if protein A is bound to a surface. However, the antibody is firmly bound to the crosslinked SCP network presenting the antigens, which means that the orientation of the Fc site is fixed, such that only a fraction of antibodies is able to bind to the protein A surface. Such hindrance is not present in classic ELISA assays where the analyte antibody is bound to a planar antigen surface from a freely dissolved state and then detected by reporter antibodies from solution. Therefore, in order to improve the detection limit of the SCP-antibody assays the elastic modulus of the SCPs should be decreased, e.g., by reducing crosslinking. This would allow larger (thermal) fluctuations of the network, and the SCP-bound antibodies would have more degrees of freedom and spatial reach to “find” protein A binding sites. Both protein A and antibodies have hydrodynamic diameters on the order of 10 nm. Thermal fluctuations of the PEG network should be of similar magnitude in order to allow sufficient probability that the orientation of the binding sites match. According to theory [29], this could be realized by polymer networks with an elastic modulus below 10 kPa, whereas the PEG-SCPs network used in this work had an elastic modulus of 60 kPa providing only fluctuations on the order of 3 nm. Another advantage of using softer PEG networks would be that the SCPs contact areas with the glass slide increase upon adhesion, thereby improving the sensitivity and detection limit of the method.

In another assay, the SCPs were charged with antibodies in presence of 50 mg mL^−1^ BSA to test the feasibility of SCP-based antibody detection from impure solutions (selectivity). The applied amount of BSA reflects typical concentrations of serum albumin in blood. Evaluation of the contact areas showed that the overall adhesion of antibody-containing SCPs was drastically reduced (factor of ten) as compared to SCPs charged with pure antibody solution. Nevertheless, the adhesion energies of SCPs prepared from antibody-containing solutions were still larger by a factor of two as compared to the negative control, SCPs treated with 50 mg mL^−1^ BSA solution containing no antibodies (Figure 6a). The significant reduction in adhesion when incubating the SCPs in presence of BSA is most likely due to non-specific binding of BSA to the SCPs, which then interferes with the specific antibody binding. Unfortunately, the mere use of PEG as carrier material for antigens is not sufficient in order to significantly reduce non-specific protein interaction, although PEG hydrogels are widely believed to be protein-repellent materials. Our finding is in line with recent studies showing that the protein-repellent properties attributed to PEG are in fact due to a protein corona that forms around PEG in blood [3]. This means that PEG does interact non-specifically with serum albumin. Therefore, in order to reduce non-specific interactions, treating the antigen/antibody surface with detergents like Tween^®^ is still required as is also the case for classic affinity assays including ELISA and the like.

Overall, we have successfully shown that the adhesion-based SCP assay allowed the detection of antibodies. For a proof-of-principle experiment, sensitivity and selectivity were not expected to match the performance of well-established methods that evolved and improved over decades like ELISA. Nevertheless, by further reducing non-specific binding and using significantly softer SCPs with increased sensitivity, further improvements in terms of sensitivity and specificity seem possible.

### 2.3. Characterization of Soil Release Polymers by SCP Adhesion Assays

**Figure 7:**
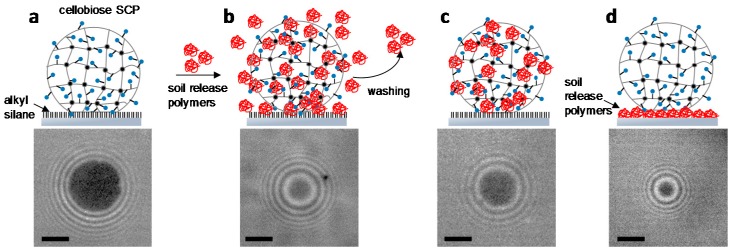
Sketches of adhesion experiments with soil release polymers (**top**) and typical SCP contact areas (**bottom**). (**a**) adhesion of bare cellobiose SCPs on hydrophobic glass as reference; (**b**) in presence of polymer samples (antiredepostion experiment); (**c**) after removal of the polymers by centrifugation and washing (antiadhesive coating experiment); (**d**) direct binding of cellobiose SCPs on polymer surfaces (direct binding experiment). Scale bars: 2 µm.

As discussed above, adhesion phenomena are ubiquitous in nature and technology. Improved understanding of these processes is best obtained by developing assays that allow mimicking and quantifying the underlying adhesion phenomena. Therefore, here we adapted the SCP adhesion assay to study soil release polymers that are used as antiadhesives and antiredeposition agents in laundry processes. The adhesion between cellobiose functionalized SCPs and a hydrophobic surface is investigated to mimic a typical laundry situation, i.e., cotton fabrics soiled with oily substances. Cellobiose is composed of two glucose units, the same building block that constitutes cellulose and used here as soluble cellulose substitute. As a mimic for an oily substance, glass slides were functionalized with a hydrophobic trichloro(octadecyl)silane by chemical vapor deposition. The contact angle of these surfaces was larger than 90° indicating the high hydrophobicity of the surface and successful silanization. Next, we studied the adhesion energy of the cellobiose SCPs on the hydrophobic surfaces by RICM contact area measurements and evaluation with the JKR model (Figure S3). The adhesion energy of the cellobiose SCP with the hydrophobic surface (Figure 7a) was used here as a reference to test the antiadhesive properties of soil release polymers. The adhesive energy was on the order of 1700 µJ m^−2^, which is in the expected range of adhesion energies in water for hydrophilic gels on hydrophobic surfaces [30]. The soil release effect was studied with four different polymers that are potential candidates as washing additive (Table 1).

Soil release polymers usually consist of two building blocks. The first one is the driver for the adsorption of the polymer on the textile, the second one provides hydrophilicity and prevents hydrophobic stains to deeply penetrate the fibers. For example, for polyester fabrics, common soil release polymers are polyesters of terephthalic acid, propylene glycol and poly(ethylene glycol). The polymer structure mimics the poly(ethylene terephthalate) (PET) garment chemistry while the PEG component hydrophilizes the surface [31]. On the other hand, for cotton textiles a copolymer consisting of a quaternary ammonium-bearing monomer and a neutral hydrophilic monomer would be better suited, where the cationic monomer acts as an anchor group for the slight anionically charged cotton fiber while the neutral comonomer provides hydrophilicity to the surface. To test the proposed mechanisms, three different experiments were conducted: (1) adhesion of cellobiose SCPs on hydrophobic surface in presence of soil release polymers in the solution (antiredeposition experiment, Figure 7b); (2) adhesion to the hydrophobic surface with cellobiose SCPs that were pre-treated and washed with soil release polymers. This experiment reflects the antiadhesive properties of soil release polymer coatings post washing (antiadhesive coating experiment, Figure 7c); (3) direct binding of cellobiose SCPs on soil release polymer surfaces (direct binding experiment, Figure 7d). A selection of the SCP adhesion data and JKR-evaluation is shown in Figure S2.

For the first experiment with the soil release polymers, a cellobiose SCP solution containing 1 mg mL^−1^ soil release polymer was allowed to adhere on the hydrophobic surfaces. In this experiment, the polymer is present in solution during adhesion and thus allows studying the activity of the soil release agents during the washing step (Figure 7b). As expected, in presence of the soil release polymers, we observed a reduction of the adhesive energy between cellobiose SCPs and the hydrophobic glass slide. The overall trend was that non-ionic hydrophilic copolymer PPT-*co*-PEG achieved the strongest reduction in adhesive energy, whereas copolymers combining cationic groups and neutral hydrophilic monomer resulted in slightly increased adhesion (Table 1). This confirms the extraordinary antiadhesive properties of the PEG part, which could be attributed to the comparatively firm hydration shell around PEG resulting in excluded volume effects [32]. Although generally considered as a hydrophilic polymer, polyacrylamide is known to show strong interactions with hydrophobic surfaces which was also confirmed in this study [33]. Overall, the significant differences in adhesion of the hydrophilic polymers investigated to hydrophobic materials are quite intriguing and still not well understood [34,35].

In the second experiment, the adhesion measurement was performed with polymer-treated SCPs but in absence of polymer in the solution during adhesion (antiadhesive coating experiment, Figure 7c). Therefore, the polymer-treated SCPs were centrifuged and washed with water to remove the polymers from the solution. As expected, the overall adhesion energies increased compared to experiments with polymers present in solution. Interestingly, most polymer samples showed comparatively large adhesion energies in the range of 1000 µJ m^−2^ close to the results for SCPs without polymers. The exception is the weakly cationic copolymer A that showed a strong reduction in adhesion energy. Interestingly, the comparative copolymer B with a higher content of cationic groups did not achieve strong reduction in adhesion energy. It could be argued that, due to its strong polyelectrolyte character this highly charged polymer shows lateral repulsion and therefore reduced binding to the SCPs. The fact that the PEG-containing polymer did not achieve a strong reduction in adhesion energy, as was the case for the first assay, could be explained by its low tendency to form a stable antiadhesive layer on the cellobiose SCPs. As a result, it was removed from the SCPs during the centrifugation and washing steps, which then resulted in adhesion energies that were comparable to the pure cellobiose on the hydrophobic glass.

Finally, the adhesion energy of cellobiose SCPs on polymer surfaces was directly measured (direct binding experiment, Figure 7d). This experiment reflects the interaction of cotton fabrics directly with the soil release polymers. Here the cationic samples showed significant binding that increased with the number of cationic groups. This could be explained by the attractive interactions of polycations with cellulose [36], which also makes them potent flocculation and retention aids for cellulose in the paper industry. The interaction was lowest in the case of the non-ionic copolymer PPT-*co*-PEG. This is expected, as PPT-*co*-PEG does not present groups that would specifically interact with cellulose. This explains the absence of an antiadhesive coating and the large adhesion energies observed in the second experiment (Figure 7c) for this polymer. Interestingly, polyacrylamide showed the largest direct binding to cellobiose SCPs, indicating that the polymer forms a stable layer on the cellobiose SCPs. However, this coating still shows strong interactions with hydrophobic materials as confirmed by the first two experiments (Figure 7b,c).

Overall, the adhesion assay with cellobiose SCPs treated with various soil release polymers indicate that nonionic poly(ethylene glycol)-containing polymer provides high antiredeposition activity when used in the aqueous phase during the washing step. Here cationic copolymers show reduced activity; however, these polymers are more likely to form a stable film on cotton fabrics in order to form an antiadhesive layer against hydrophobic materials post washing. This confirms the generally believed mechanism of polymeric soil release: for cellulose-specialized soil release polymers, cationic groups of copolymers can bind to the slightly negatively charged cellulose, whereas the neutral part provides hydrophilicity and steric repulsion to the surface in order to reduce adhesive interactions.

## 3. Conclusions

In summary, a novel adhesion assay based on soft PEG hydrogel particles was established to investigate specific interactions of antibodies and antigens as well as cellobiose on hydrophobic surfaces in presence of soil release polymers. We tested the applicability of these assays in two commercially relevant areas, medical diagnostics of antibodies and detergent additives. In both cases, the adhesion assay could provide statistically significant amount of data in a short timescale compared to classic adhesion assay by means of AFM or surface force apparatus. It should be taken into account, however, that the adhesion method still requires the synthesis of the soft hydrogel particles for sensing, whereas in the case of more established methods like AFM the basic sensors (e.g., silicon cantilevers) are already available. In addition, regarding antibody detection, the SCP adhesion-based assay is still inferior in terms of sensitivity compared to established methods like ELISA. The sensitivity of antibody detection via adhesion of hydrogels sensors could be principally hampered due to the requirement of having the biomolecules properly oriented on the adhering surfaces. One of the strengths of the adhesion assays with soft, gel-like sensor particles is that it can mimic adhesive processes in nature and technology. This was in particular useful for the analysis of the soil release polymers, where several types of adhesion experiments could be established to decipher the mode of action of soil release polymers. It was found that the nonionic PEG-containing polymer is very active in antiredeposition of fats in aqueous media, whereas polymers containing cationic groups are more suited as antiadhesive coating on cellulose. The work shows that the presented SCP-RICM technique is well suited to study a large range of adhesion phenomena in nature and technology.

## 4. Experimental Section

### 4.1. Materials

Soil release polymer samples were provided by Henkel AG & Co KGaA and used without further purification (purity > 95%) The fluorescein antibody (polyclonal, type IgG) was produced by Rockland Immunochemicals (Limerick, PA, USA) and obtained from Biomol GmbH (Hamburg, Germany). All other chemicals were obtained from Sigma-Aldrich (Darmstadt, Germany). All water used here was produced by purification system with a resistivity higher than 18.2 MΩ∙cm at 25 °C and UV treatment to break down organic impurities.

### 4.2. Soft Colloidal Probe Preparation

PEG SCPs were synthesized by crosslinking a dispersion of PEG-dAAm macromonomer droplets in a similar manner as described previously [8,14]. PEG-dAAm (Mn = 8000 Da) (50 mg, 6.3 µmol) was dispersed in a 1 M sodium sulfate/PBS solution (10 mL). Crotonic acid (400 µmol) were added to adjust the elastic modulus of the SCPs to about 30 kPa. Then the UV photoinitiator Irgacure 2959 (1 mg, 4.5 µmol) was added to the dispersion and vigorously shaken and photopolymerized under UV light. The PEG SCPs were washed with water and stored in water. The resulting particles were 10–70 µm in diameter. Next, the PEG SCPs were grafted with crotonic acid (CA) [21]. Briefly, water was exchanged by ethanol and benzophenone (250 mg, 1.4 mmol) and CA (1.5 g, 17.4 mmol) were added. Then the mixture was flushed with nitrogen for 30 s and irradiated with UV light for 900 s. The PEG-CA SCPs were washed with ethanol. FITC functionalized SCPs were prepared by first reacting the PEG-CA SCPs in 0.1 M 2-(*N*-morpholino)ethanesulfonic acid (MES) buffer pH 5.5 containing 0.1 M *N*-Hydroxysuccinimide and 0.1 M *N*-Ethyl-*N*’-(3-dimethylaminopropyl)carbodiimide (EDC). Next, the activated PEG-CA SCPs were centrifuged, washed and added to a 0.5 mg L^−1^ BSA solution in 0.1 M phosphate buffer (pH 8.0). Finally, 30 µL of a 0.5 mg mL^−1^ solution of FITC in DMSO were added to 2 mL of BSA-SCPs to form FITC-BSA SCPs followed by centrifugation and washing. Cellobiose functionalized SCPs were prepared by incubating the PEG-CA SCPs in 0.1 M MES buffer (pH 4.5) containing 0.1 M EDC and 1 mg mL^−1^ cellobiose for 2 h followed by washing and washing.

### 4.3. SCP Characterization

AFM force spectroscopy with a NanoWizard 3 system (JPK instruments AG, Berlin, Germany) was performed to determine the elastic modulus of the microparticles. As AFM probe a glass bead with a diameter 4.75 µm (cellobiose SCPs) or 10 µm (FITC-BSA SCPs) was glued with an epoxy glue onto a tipless, non-coated cantilever (spring constant 0.32 N m^−1^; CSC12, NanoAndMore GmbH, Wetzlar, Germany). Several force curves were recorded for different SCPs and analyzed with an appropriate contact model developed by Glaubitz et al. [23].

### 4.4. Surface Preparation

Round glass coverslips (35 mm #1, Menzel Gläser, Braunschweig, Germany) were cleaned in a mixture of ammonia hydrogen peroxide (30%) and water (1:1:5, RCA protocol) at 70 °C. Protein A coated surface, glass slides were immersed in 0.5 mg mL^−1^ protein A (PBS pH7.4), flushed with RICM measurement solution (PBS pH7.4). For soil release polymer coating, glass slides were immersed in a mixture of 182.4 mL ethanol, 9.6 mL water, 192 µL acetic acid, and 1920 µL GLYMO, shaken for 120 min, flushed with ethanol, followed by annealing for 120 min at 90 °C. Before RICM measurement, the GLYMO slides were immersed in measurement solution (PBS pH7.4) containing soil release polymers a concentration of 1 mg mL^−1^, shaken for 60 min, and flushed with RICM measurement solution. Hydrophobic surfaces mimicking contact to oily soils were prepared by exposing cleaned coverlips to an atmosphere of trichloro(octadecyl)silane; 5 mL of the silane were cast in a glass petri dish and placed in a vacuum desiccator with the coverslips. After adjusting a pressure of 20 mbar by a vacuum pump, the valves were closed and the coverslips were left overnight in the desiccator. Finally, the surfaces were rinsed with isopropanol and water followed by curing for two hours at 150 °C in a drying oven.

### 4.5. Determination of Functionalization Degree via TBO Titration 

A quantity of 0.5 mL carboxylic group functionalized SCPs dispersion was washed with ethanol and dried under vacuum at 50 °C for 5 h until constant weight was reached. 1 mL toluidine blue O (TBO) aqueous solution with a concentration of 312.5 µM at pH 10–11 was added to the dry SCPs and shaken in the dark overnight to stain the SCPs. The stained SCP dispersion was centrifuged for 30 min at 4400 rpm. 0.3 mL of the supernatant was diluted to 2 mL with water. The absorbance at 633 nm of this solution was measured by UV-VIS spectroscopy and compared to the absorbance of a TBO standard (312.5 µM TBO in aqueous solution at pH 10–11 and 1.7 mL). The carboxylic group functionalization degree of this group of SCPs was calculated with the following equation $D_{CGF}=N_{R}(1-A_{S}/A_{R})/W_{Dry}$, where *D_CGF_* is the carboxylic group functionalization degree, *A_S_* and *A_R_* is the UV-VIS absorbance of sample and reference, *W_Dry_* is the dry weight of 0.5 mL SCPs, *N*_R_ is the amount of TBO in the reference in units of µmol. For each group of SCPs, the TBO titration experiment was repeated three times and the average carboxylic group functionalization degree of the three experiments was used as the carboxylic group functionalization degree of this group of SCPs.

### 4.6. Reflection Interference Contrast (RICM) Measurements

RICM on an IX 73 inverted microscope (Olympus, Tokyo, Japan) was used to obtain the contact area between the SCPs and the glass coverslip surfaces. For illumination, an Hg-vapor arc lamp was used with a green monochromator (546 nm). An UPlanFL N 60×/0.90 dry objective (Olympus Corporation, Japan), and uEye CMOS camera (IDS Imaging Development Systems GmbH, Obersulm, Germany) were used to image the RICM patterns. To conduct the JKR measurements of the adhesion energies, both the contact radius and the particle radius were measured. Images with RICM patterns were read out using self-written image analysis software, contact areas and particle profiles were evaluated using scripted peak finding algorithms (IgorPro Wavemetrics, Lake Oswego, OR, USA).

## Figures and Tables

**Table 1:** Soil release polymer samples and adhesion energy from the three different assays. ^a^ Direct binding experiment between cellobiose SCP and hydrophobic surface. ^b^ Polymer was physisorbed on the glass/glymo slide.

Polymer	Adhesion Energies [µJ m^−2^]		
	Antiredeposition	Antiadhesive Coating	Direct Binding
none/reference		1700 ^a^	
Poly(propylene terephthalate)-*co*-Poly(ethylene glycol) (nonionic) PPT-*co*-PEG	41	1038	38 ^b^
Copolymer A: cationic/neutral hydrophilic ratio 22:78	163	480	91
Copolymer B: cationic/neutral hydrophilic ratio 70:30	306	1020	126
Poly(acrylamide)	1142	1429	243

## Supplementary Materials

Supplementary materials can be found at www.mdpi.com/1422-0067/3/3/31/s1. Figure S1: A typical AFM indentation curve for SCP elastic modulus determination. The example shows a FITC-BSA SCP after functionalization. Red circles represent data points; blue line represents fits according to the equation above; Figure S2: Typical JKR plots (contact radii vs. SCP size) for cellobiose SCPs on alkylsilane surfaces: direct binding experiment for cellobiose adhering to alkylsilane surface (filled circles), antiadhesive coating experiment for copolymer A (cross symbols) and PPT-*co*-PEG (nonionic). Note the strongly reduced contact areas for the cellulose specialized soil release agent copolymer A; Figure S3: Schematic drawing of the RICM principle.
