# Supplementary material for: Hydrogel Microparticles as Sensors for Specific Adhesion: Case Studies on Antibody Detection and Soil Release Polymers
Source: Gels. 2017 Aug 8;3(3):31. doi: 10.3390/gels3030031 (PMC6318626; doi:10.3390/gels3030031)
Supplement: Supplementary file 1 [file gels-03-00031-s001.pdf]

Supporting Information

Hydrogel Microparticles as Sensors for Specific Adhesion: Case Studies on Antibody Detection and Soil Release Polymers

Alexander Klaus Strzelczyk, Hanqing Wang, Andreas Lindhorst, Johannes Waschke, Tilo Pompe, Christian Kropf, Benoit Luneau and Stephan Schmidt

S1 Determination of the SCPs elastic modulus

Force spectroscopy with a NanoWizard 3 AFM system was performed to determine the elastic modulus of the microparticles. Therefore a glass bead with a diameter of 4.75 µm (cellobiose SCPs) or 10 µm (FITC-BSA SCPs) was glued with an epoxy glue onto a tipless, non-coated cantilever (spring constant 0.32 N/m; NanoAndMore GmbH). Several force curves were recorded from different particles and analyzed with the novel contact model developed by Glaubitz et al [23]. The model considers deformation of the object at two sites: the indentation site of the AFM probe and at the contact with the solid support. The respective deformation (δ)–force (F) dependence reads:

$$\delta(F) = \left(\frac{3F}{4E}\cdot\frac{1-\upsilon^2}{R_{AFM}^{\frac{1}{2}}}\right)^{\frac{2}{3}} + \left[\frac{3(1-\upsilon^2)\left(F + 6W\pi R_{SCP} + \sqrt{12W\pi R_{SCP}F_c(6W\pi R_{SCP})^2}\right)}{4E\cdot R_{SCP}^{\frac{1}{2}}}\right]^{\frac{2}{3}} - \left[\frac{9W\pi(1-\upsilon^2)}{E}\right]^{\frac{2}{3}}\cdot R_{SCP}^{\frac{1}{3}}$$

where E is the elastic modulus of the indented SCP, R_{SCP} its radius, υ the Poisson ratio of the SCP, W the SCP adhesion energy with the support surface and R_{AFM} the radius of the indenter. The Poisson ratio was assumed to be 0.5 (volume conservation upon indentation). E and W were free fit parameters. The elastic moduli of SCPs were on the order of 60 kPa and their surface energy varied only marginally between 10 and 15 µJ/m^2 for the different fits. Below four typical deformation (δ)–force (F) data (red circle) and fit (blue line) for a FITC-BSA SCP:

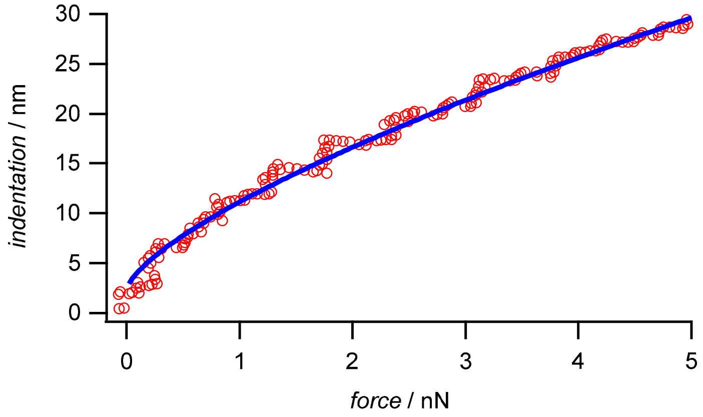

Figure S1. A typical AFM indentation curve for SCP elastic modulus determination. The example shows a FITC-BSA SCP after functionalization. Red circles represent data points; blue line represents fits according to the equation above.

S2 Typical JKR Plots for soil release polymers

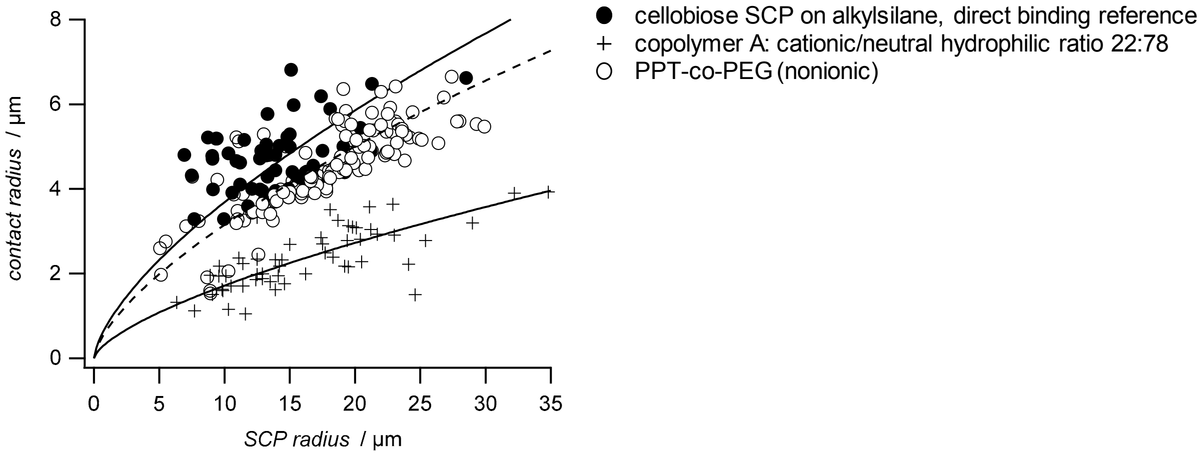

Figure S2. Typical JKR plots (contact radii vs. SCP size) for cellobiose SCPs on alkylsilane surfaces: direct binding experiment for cellobiose adhering to alkylsilane surface (filled circles), antiadhesive coating experiment for copolymer A (cross symbols) and PPT-co-PEG (nonionic). Note the strongly reduced contact areas for the cellulose specialized soil release agent copolymer A.

S3 Reflection Interference Contrast Microscopy (RICM) measurements

Setup

RICM on an inverted microscope (Olympus IX73) was used to obtain the contact area between the microparticles and a hard glass surface. For illumination a monochromatic (530 nm) collimated LED (Thorlabs, Germany, M530L2-C1) was used. An Olympus 60 x NA 1.35 oil-immersion objective (UPLSAPO60XO/1,35 U Plan S Apo), additional polarizers and a quarter waveplate (Thorlabs, Germany) to avoid internal reflections and

a monochrome CMOS camera (UI-3360CP-M-GL, IDS Germany) were used to image the RICM patterns. To conduct the JKR measurements, both the contact radius (in RICM mode) and the particle radius (in transmission mode) were measured. Image acquisition was done using µManager (v1.4.16), data analysis was done using the image analysis software Image-J (v1.48) and the mathematical software IgorPro (v6.38, Wavemetrics, USA).

Determination of the contact radius

RICM was used to measure the contact radius formed by the SCPs resting on the polymer surface (Figure S8a). Polarized light waves reflected from the upper glass surface (I_1) and the surface of the bead (I_2) interact to create an interference image. The intensity at a given position in the image depends on the separation $h(x)$ between the two surfaces: $I(x) = I_1 + I_2 + 2 \cdot \text{sqrt}(I_1 \cdot I_2) \cos[2k \cdot h(x) + \pi]$, where $k = 2\pi n/\lambda$, and n and λ are the index of refraction of water and the wavelength of the monochromatic light, respectively. In order to detect the interference pattern, stray light was reduced by an 'antiflex' technique. This is accomplished by crossed polarizer and analyzer filter with a λ/4-plate placed between the objective lens and the analyzer [11].

Practical note: Although it is generally recommended to use the antiflex optics with polarization methods to avoid stray light generated in the microscope, we observed only little improvement in image contrast when using the antiflex setup. RICM images could be readily taken without polarizer, analyzer and quarter wave plate. This is possibly due to the rigorous use of antireflective lenses in the microscope and Thorlabs components.

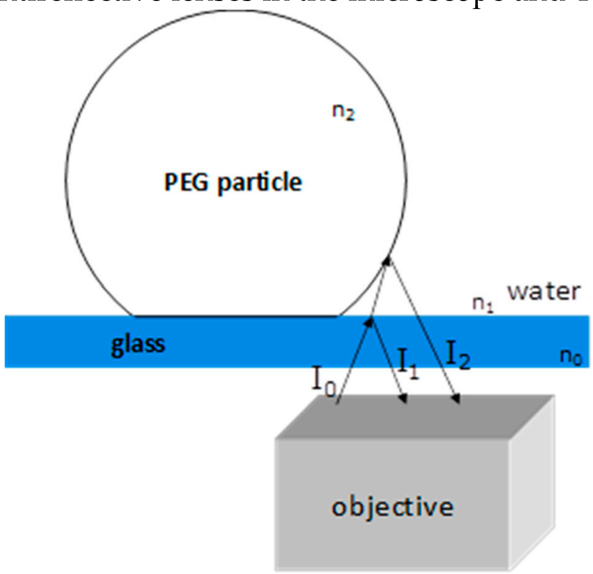

Figure S3a. Schematic drawing of the RICM principle.

Correction Factors

For analysis of the intensity distribution correction factors must be determined for finite aperture and geometry effects. To obtain the correction factors, we imaged hard, non-deformable glass beads on a glass surface in RICM mode (Figure S3a) with a known size.

We recorded 5 glass beads with a diameter in the range of 10-20 µm and extracted the intensity profile. Using the profiles, we reconstructed the shape of the beads and compared it to the known spherical shapes of the glass beads (glass bead radius R measured by light microscope), and determined the correction factors, see Pussak et al [14].

Contact radius determination

To determine the contact radius *a* of the SCP on the polymer surface we reconstructed the height profile of the particles from the RICM images (see Figure S3b). This was done by determining the lateral $x(i)$ positions of the i-th minima and maxima by a self-written IgorPro procedure (Wavemetrics, USA). Next, the vertical position $y(i)$ of the maxima and minima were determined by

$$y(i) = \frac{i\lambda}{4n} + c_i ,$$

where n is the refractive index, λ the wavelength and c_i the correction factors. The height profile was then reconstructed by plotting $y(i)$ vs $x(i)$ and fitting the data by a circle equation representing the assumed shape of the SCP:

$$y(x) = y_0 + \sqrt{R^2 - x^2} .$$

where R is the independently measured SCP radius and y_0 the vertical shift of the SCP center due to flattening of the SCP upon adhesion. The fit with y_0 as the only free fit parameter intersects with the x-axis and gives the contact radius *a*.

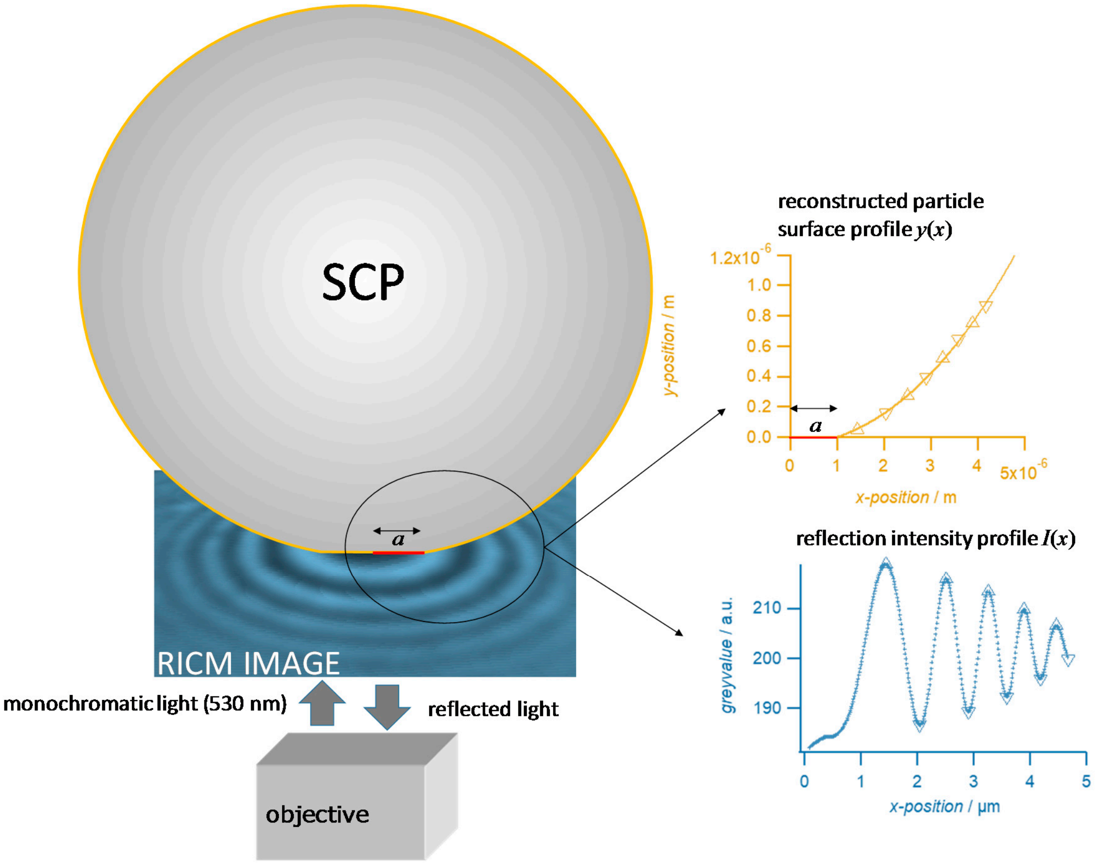

Figure S3b Left: schematic representation of the measurement setup. Bottom right: actual intensity profile of an adherent SCP showing 5 minima and 5 maxima. Top right: reconstructed surface profile of the SCP and the contact radius a at the intersection of the profile at $y = 0$.
